# The repertoire of protein-sulfatide interactions reveal distinct modes of sulfatide recognition

**DOI:** 10.3389/fmolb.2022.1080161

**Published:** 2022-11-30

**Authors:** Daniel G. S Capelluto

**Affiliations:** Protein Signaling Domains Laboratory, Department of Biological Sciences, Fralin Life Sciences Institute, Center for Soft Matter and Biological Physics, Virginia Tech, Blacksburg, VA, United States

**Keywords:** sulfatide, α-synuclein, disabled-2, mesencephalic astrocyte-derived neurotrophic factor, myeloid differentiation factor-2, cluster of differentiation 1, glycolipid-transfer protein, cardiotoxin

## Abstract

Sulfatide is an abundant glycosphingolipid in the mammalian nervous system, kidney, trachea, gastrointestinal tract, spleen, and pancreas and is found in low levels in other tissues. Sulfatide is characterized by the presence of a sulfate group in the hydrophilic galactose moiety, with isoforms differing in their sphingosine base and the length, unsaturation, and hydroxylation of their acyl chain. Sulfatide has been associated with a variety of cellular processes including immune responses, cell survival, myelin organization, platelet aggregation, and host-pathogen interactions. Structural studies of protein-sulfatide interactions markedly advanced our understanding of their molecular contacts, key-interacting residues, orientation of the sulfatide in its binding site, and in some cases, sulfatide-mediated protein oligomerization. To date, all protein-sulfatide interactions are reported to display dissociation constants in the low micromolar range. At least three distinct modes of protein-sulfatide binding were identified: 1) protein binding to short consensus stretches of amino acids that adopt α-helical-loop-α-helical conformations; 2) sulfatide-bound proteins that present the sulfatide head group to another protein; and 3) proteins that cage sulfatides. The scope of this review is to present an up-to-date overview of these molecular mechanisms of sulfatide recognition to better understand the role of this glycosphingolipid in physiological and pathological states.

## 1 Introduction

**FIGURE 1:**
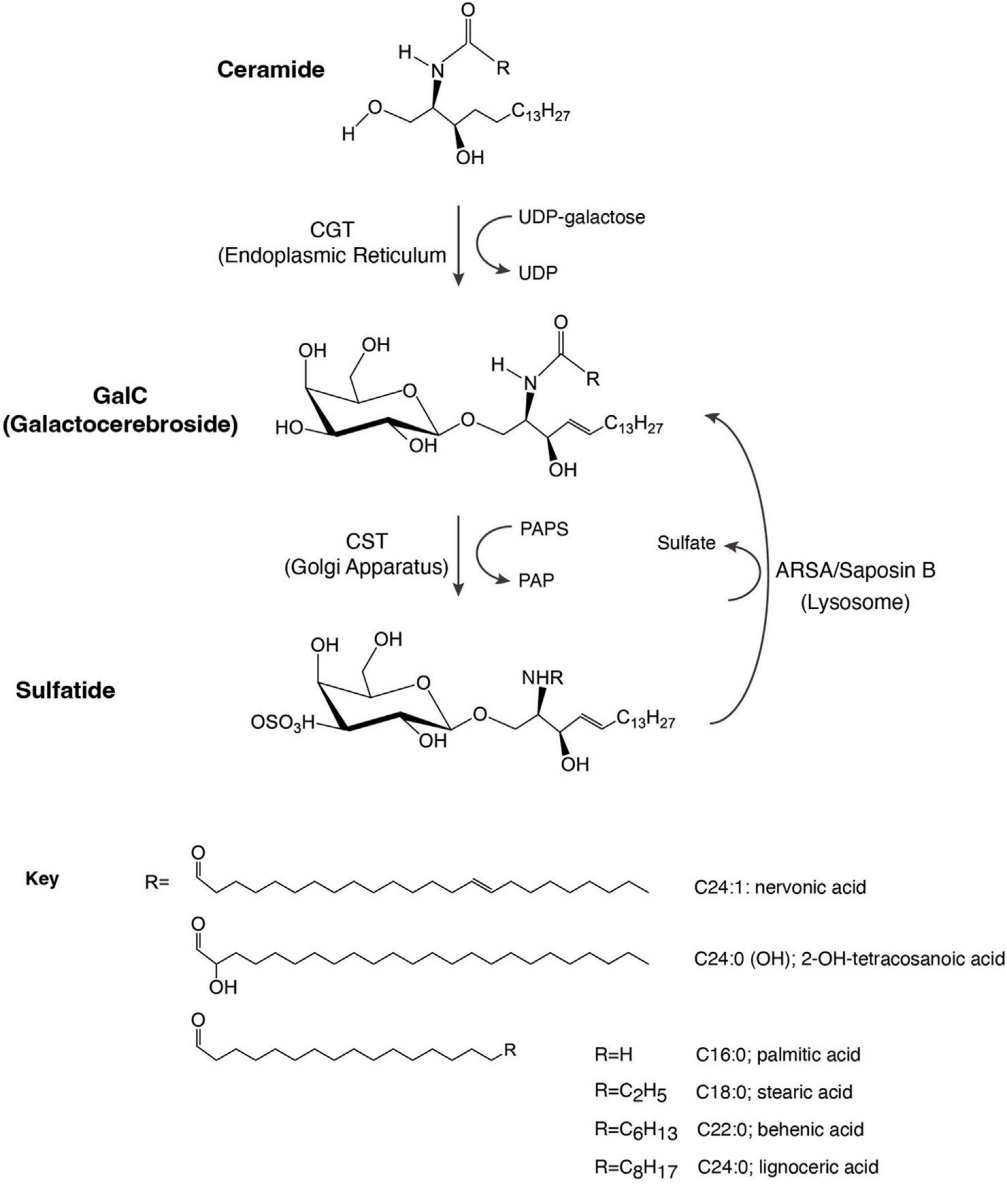
Schematic representation of sulfatide synthesis and degradation. CGT, galactosyl transferase; CST, cerebroside sulfotransferase; GalC, galactosylceramide; PAP, 3′-phosphoadenosine 5′-phosphate; PAPS, 3′-phosphoadenosine 5′-phosphosulfate; ARSA, arylsulfatase.

Sulfatide (3-*O*-sulfogalactosylceramide or SM4) is a sphingolipid found on the surface of all eukaryotic cells. Through its negatively charged head group, sulfatide interacts with adhesive receptors, coagulation factors, innate immunity receptors, and extracellular proteins (Xiao et al., 2013). In eukaryotic cells, sulfatide isoforms display differences in fatty acid lengths as well as degrees of unsaturation and hydroxylation. For example, mice kidney has seventeen sulfatide species (Nakashima et al., 2022), whereas human renal cell carcinoma has 52 sulfatide species (Jirasko et al., 2017). Although their physiological functions are unclear, some studies revealed predominantly tissue-dependent sulfatide species. The C18:0 and hydroxylated forms are found in the formation of oligodendrocytes, whereas longer fatty acid tails are observed in mature cells (Hirahara et al., 2017). It is possible that such increases in the tail length would alter their cell membrane fluidity and, thus, the oligodendrocyte function and development. Other cells, such as pancreatic cells, contain shorter C16:0 sulfatide species (Buschard et al., 2006). Deletion of the cerebroside sulfotransferase (CST) gene, which encodes the enzyme that catalyzes the last step in the biosynthesis of sulfatide (Figure 1), leads to a lack of sulfatide in mouse brain tissue (Honke et al., 2002). In this deletion, mice tremor by 4–6 weeks of age, are paralyzed by about 1 year of age, and die prematurely at about 15 months of age. These results indicate that sulfatide may not be critical for the development of myelin but impacts myelin maintenance.

The role of sulfatide in membrane organization has been revealed from studies using phase-selective fluorescent probes (Bjorkqvist et al., 2008). In the absence of sphingomyelin, sulfatide segregates from the phosphatidylcholine-enriched phase and cholesterol; however, in the presence of sphingomyelin, sulfatide shares the same phase with both sphingomyelin and cholesterol and remains excluded from the phosphatidylcholine-enriched phase (Bjorkqvist et al., 2008). Despite this observation, sphingolipids (such as sulfatide) and cholesterol show complementary geometry, which may favor their interactions independently of the presence of sphingomyelin. Sphingolipids have a large polar region and a small nonpolar region, whereas cholesterol displays a conical shape with a small head group and a large nonpolar region. Thus, it has recently been proposed that cholesterol acts as both a molecular wedge and as cement through hydrophobic interactions with the narrow sphingolipid nonpolar region; otherwise sphingolipids would spontaneously form micellar structures (Azzaz et al., 2022).

Sulfatide and galactosylceramides (GalCs) are the major glycosphingolipids in myelin, representing about 4 and 23% of the total lipid content, respectively (Ozgen et al., 2016). Their critical role is to facilitate the lateral carbohydrate-carbohydrate interactions and organization of the lipid raft, a dynamic membrane region required for signal transduction, protein trafficking, and membrane remodeling (Boggs et al., 2010). Unlike GalCs, the negative charge on the sulfatide head group perturbs its packing arrangement on membranes due to lateral charge repulsion (Saxena et al., 2000). A reduction in sulfatide levels has been measured in lipid rafts isolated from the frontal cortex of Parkinson’s disease patients (Fabelo et al., 2011) and in post-mortem brain analysis of patients with pre-clinical or early-stage Alzheimer’s disease (Cheng et al., 2013). Accumulation of sulfatides can also trigger a variety of diseases. For example, metachromatic leukodystrophy (MLD) is a lysosomal storage syndrome that is promoted by deficiency of the enzyme arylsulfatase (ARSA; EC 3.1.6.8) and, in some cases, because of defects in the activity of the ARSA cofactor saposin B (Cesani et al., 2016), both of which are required for sulfatide turnover (Figure 1). In either of these scenarios, the central and peripheral nervous system, liver, kidney, and gall bladder display an increase in sulfatide levels. Levels of specific sulfatide species can also lead to disorders, such as in the Alport syndrome where the sulfatide species d18:2/24:0 and d18:2/16:0 are elevated in mice renal tubes, suggesting that these species may be linked to the formation of tubulointestinal fibrosis, which is associated with the disease (Gessel et al., 2019). ARSA variants are also linked to the Parkinson’s diseases, in which the enzymes serve as chaperones for α-synuclein (Vestermark et al., 1987).

## 2 Synthesis and degradation of sulfatides

Sulfatide synthesis begins in the endoplasmic reticulum (ER) where 2-hydroxylated ceramides and non-hydroxylated ceramides are substrates for UDP-galactose in a reaction mediated by the ceramide galactosyltransferase (CGT, EC 2.4.1.45), leading to the formation of GalC (Figure 1). GalC is delivered through vesicular trafficking to the Golgi apparatus, serving as a substrate for CST (EC 2.8.2.11), which transfers a sulfonate group from the donor substrate, 3′-phosphoadenosine 5′-phosphosulfate, to the hydroxyl group of GalC to produce sulfatide. Once produced, sulfatide can be distributed to the outer leaflet of the cell membrane or it can be released to the extracellular space (Takahashi and Suzuki, 2012).

Lysosomal catabolism of sulfatide is mediated by the co-action of ARSA and the sphingolipid activator protein-1 or saposin B, producing GalC (Figure 1). Indeed, saposin B deficiency impairs ARSA to properly interact with sulfatide (Fischer and Jatzkewitz, 1977; Inui et al., 1983).

## 3 Distinct sulfatide-binding principles

### 3.1 Consensus α-helical-loop-α-helical domains

#### 3.1.1 Disabled-2

Disabled 2 (Dab2) is a modular protein involved in a variety of cellular processes, including endocytosis and modulation of platelet function (Finkielstein and Capelluto, 2016). In humans, the Dab2 gene is localized to chromosome 5p13 and encodes a 96 kDa protein. Alternatively spliced Dab2 also leads to the expression of a 67 kDa protein (Chetrit et al., 2009). Dab2 functions as an adaptor due its modular architecture, which includes a phosphotyrosine-binding (PTB) domain, multiple clathrin-binding sites, NPF and DPF motifs, and the presence of an SH3 domain at the C-terminus (Finkielstein and Capelluto, 2016).

Dab2 negatively controls platelet aggregation through the modulation of both inside-out and outside-in signaling pathways. In inside-out signaling, cytosolic Dab2 is phosphorylated by the protein kinase C at S24, promoting Dab2 membrane targeting, where it associates with the intracellular region of the αIIbβ3 integrin receptor, leading to the inhibition of fibrinogen-mediated platelet adhesion (Huang et al., 2004). Association of Dab2 to the inner leaflet of the plasma membrane is likely facilitated by the binding of the PTB domain to phosphatidylinositol 4,5-bisphosphate [PtdIns(4,5)P_2_] (Howell et al., 1999; Alajlouni et al., 2011). Indeed, accumulation of PtdIns (4,5) P_2_ in the inner leaflet membrane of activated platelets (Racaud-Sultan et al., 1993) plays multiple roles such as in platelet shape (Hartwig et al., 1995) and spreading (Heraud et al., 1998).

Through platelet outside-in signaling, ligand-receptor complexes promote platelet spreading, stabilization of platelet adhesion and aggregation, secretion of intracellular granules, and retraction of clots (Rivera et al., 2009). Dab2 displays a role in outside-in signaling by binding to both the extracellular region of the α_IIb_ subunit of the integrin receptor and to membrane sulfatide (Welsh et al., 2011). Dab2 reaches the extracellular surface through a secretory mechanism that involves platelet α-granules (Huang et al., 2006). As a counterpart, Dab2 is a substrate of the platelet agonist thrombin, which cleaves Dab2 at its N-terminus (Huang et al., 2006); this reaction may be prevented when Dab2 is associated with sulfatide (Drahos et al., 2009). Also, binding of Dab2 to sulfatide outcompetes P-selectin association to sulfatide (Welsh et al., 2011) as a mechanism to control the extent of P-selectin-mediated platelet aggregation (Merten et al., 2000).

**FIGURE 2:**
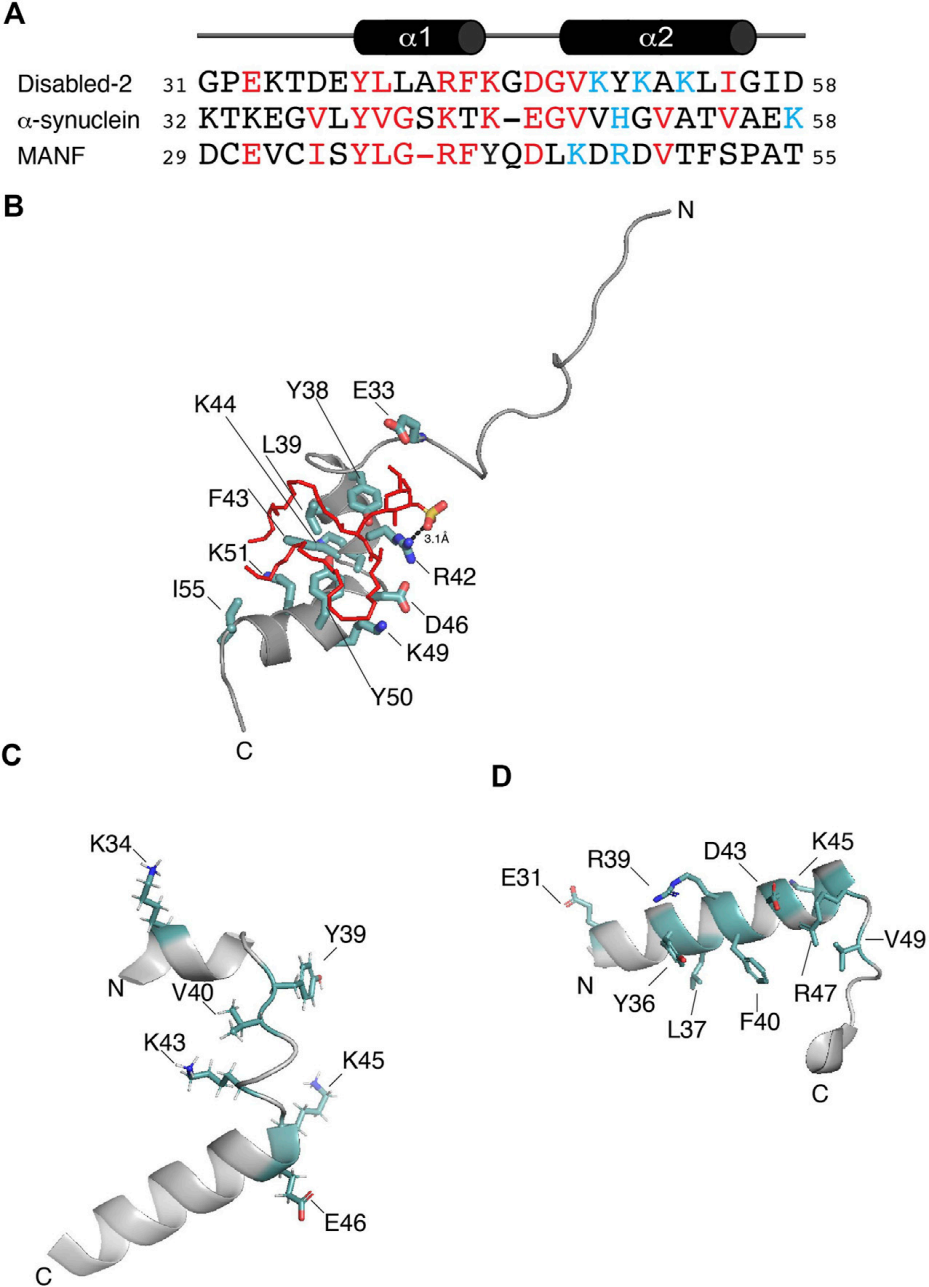
**(A)** Protein sequence alignment of the potential sulfatide-binding domains of Disabled-2, α-synuclein, and MANF, all of which are of human origin. Conserved residues are in red, whereas residues proposed to be in the polybasic region are colored in light blue. Sequence alignment was generated using COBALT and the default advanced parameters, except for the gap penalties, which are -3 (opening) and -3 (extension). Secondary structure of Dab2 SBP is depicted on top of the alignment. **(B)** Docking of sulfatide into human Dab2 SBM (PDB: 2LSW). Residues critical for sulfatide binding are colored in light teal. **(C)** Human α-synuclein (residues 32–58; PDB: 1XQ8) showing the residues involved in GM1 binding as well as residues (all in light teal) that are conserved in the alignment shown in **(A)**. **(D)** Human MANF (residues 29–55; PDB: 2W51) showing the proposed sulfatide-binding site involving the most conserved residues (light teal) shown in **(A)**.

Dab2 was first reported to bind sulfatide through its N-terminal region, including its PTB domain (Drahos et al., 2009). Mutagenesis in Dab2 residues K25, K49, K51, and K53 reduces sulfatide association, affecting Dab2-mediated platelet aggregation (Drahos et al., 2009). Later, structural studies identified the minimal region within Dab2 that binds sulfatide (residues 24–58 of human Dab2) with a dissociation constant (*K*_D_) of ∼30–50 μM (Table 1), inhibiting platelet-platelet interactions (Xiao et al., 2012; Song et al., 2020). This region, which we define here as the Dab2 sulfatide-binding motif (SBM), contains an N-terminal disordered region followed by two short α-helices, which are involved in membrane insertion (Xiao et al., 2012). Thus, Dab2 SBM is a prototype of a canonical sphingolipid-binding domain (SBD) characterized by an α-helical structure with a central aromatic residue and a basic residue found at each end (Fantini, 2003). Interestingly, SBDs are also found in viral and other amyloidogenic proteins (Fantini et al., 2002). One of the features of SBDs is that they display low amino acid sequence homology (Fantini et al., 2002) (Figure 2A). Backbone dynamics studies of a peptide representing Dab2 SBM indicate that its N- and C-termini are flexible, whereas a rigid structure is found within the α-helical regions, which is not altered by sulfatide (Song et al., 2020). Combined structural and molecular dynamics simulation (MDS) studies refined the sulfatide-binding site in Dab2 SBM. Residues located upstream and on the first α-helix are engaged in sulfatide head group contacts with Dab2 R42 playing a primary role, although other residues nearby, including E33, Y38, and K44 are required for sulfatide association (Figure 2B) (Song et al., 2020). Replacement of R42 with lysine reduces association to sulfatide-enriched lipid bilayers and impairs inhibition of P-selectin surface location in platelets (Song et al., 2020). Thus, the stereochemistry of R42, and not necessarily the positive charge in it, is critical for sulfatide binding. The second α-helix of Dab2 SBM, which includes a BXBXBX (B, basic; X, any residue) motif, is involved in binding to sulfatide hydrophobic chains (Song et al., 2020). The hydrophobic patch in the second α-helix of Dab2 SBM is built by residues Y50, L54, and I55, but is also decorated by the polar residues K49, K51, and K53 (Xiao et al., 2012). The putative function of the lysine residues in the second α-helix is consistent with the dual role of lipid binding through lysine residues; that is, electrostatic contacts through their ε amino groups and hydrophobic contacts *via* their flexible hydrocarbon chains (Hoernke et al., 2012). Interestingly, other cell adhesive proteins, such as laminins, selectins, and thrombospondins employ their basic clusters for sulfatide binding (Ishizuka, 1997), suggesting common binding mechanisms.

**TABLE 1:** Summary of the properties of selected sulfatide-binding proteins.

Protein	Sulfatide-dependent function	Number of bound sulfatide	Sulfatide orientation head group/acyl chains	Sulfatide-bound oligomeric state	KD for sulfatide (μM)	References
DAB2	Regulation of platelet aggregation	1	in/in	Monomer	30–50	Drahos et al. (2009), Song et al. (2020)
TLR4-Md-2	Sterile inflammation/Suppress LPS-mediated inflammation	6	out/in	Heterotetramer	unknown	Su et al. (2021)
CD1A	Sulfatide (auto-antigen) presentation	1	out/in	Monomer	22	Paterson et al. (2021)
CD1A-TCR	May or may not block TCR depending on TCR isoform	1	out/in	Heterodimer	10–22	Wegrecki et al. (2022)
CD1D	Sulfatide (auto-antigen) presentation	1	out/in	Monomer	unknown	Zajonc et al. (2005)
CD1D-TCR	Activation of type I and II natural killer T cells	1	out of CD1d; towards TCR/in CD1d	Heterodimer	18–26	Patel et al. (2012), Luoma et al. (2013), Stax et al. (2017)
GLTP	Intermembrane trafficking of sulfatide	1	in/in	Dimer	unknown	Samygina et al. (2011)
CTX	Host membrane pore formation	1	in/out	Dimer/Tetramer	33	Tjong et al. (2007)

#### 3.1.2 α-synuclein

Cytosolic accumulation of the protein α-synuclein, which leads to the formation of inclusions known as Lewy bodies and Lewy neurites, is a hallmark feature of Parkinson’s disease (Fields et al., 2019). α-synuclein is a 14 kDa intrinsically disordered protein with an N-terminal lipid-binding membrane-dependent α-helix, a non-amyloid component, and an acidic C-terminal domain. The lipid-binding α-helix is composed of seven 11-residue repeats, which are thought to insert into membranes to alter their curvature (Davidson et al., 1998). Lipids are known to form a complex with α-synuclein, which co-aggregate and promote α-synuclein misfolding (Musteikyte et al., 2021). Using surface pressure measurements of sphingolipid monolayers, Fantini and Yahi reported that α-synuclein can bind a wide range of sphingolipids, including sulfatide (Fantini and Yahi, 2011). Accordingly, the authors identified a consensus SBD in α-synuclein, which displays common properties with Dab2 SBM and other sphingolipid-binding proteins (Figure 2A). α-synuclein is composed of a canonical SBD, with residues 34–45 representing the minimal unit capable of recognizing monosialodihexosylganglioside (GM3) and other sphingolipids including sulfatide (Fantini and Yahi, 2011). Interestingly, mutations within this region such as A30P, E46K, H50Q, G51D, and A53T, are familial forms of Parkinson’s disease (Polymeropoulos et al., 1997).

Studies carried out using MDS indicate that α-synuclein, primarily through Y39, simultaneously binds two molecules of GM3, which is the preferred sphingolipid for the protein (Fantini and Yahi, 2011). The Y39 residue in α-synuclein (Figure 2C) is found at the interface between the polar and nonpolar regions of GM3, mediating the insertion of the protein into the membrane. In comparison, Dab2 SBM Y38, which is relevant for sulfatide interactions, is positioned near by the head group of the sphingolipid as deduced from MDS (Song et al., 2020) and, based on NMR paramagnetic studies, likely lays underneath of the lipid monolayer (Xiao et al., 2012). α-synuclein K34 also mediates interactions with GM3 (Fantini and Yahi, 2011). Dab2 SBP K34, on the other hand, seems to be dispensable for sulfatide interactions (Song et al., 2020). Αlthough not required for GM3 interactions, α-synuclein K43 may play a similar function to that reported for Dab2 R42 in sulfatide interactions. In addition, given that the Dab2 SBP R42K mutation decreased sulfatide binding, α-synuclein K43, instead of R43, might also reduce preference for the sphingolipid.

#### 3.1.3 The mesencephalic astrocyte-derived neurotrophic factor

The mesencephalic astrocyte-derived neurotrophic factor (MANF) is localized at the lumen of the ER where it functions as a secretory factor (Al-Aghbar et al., 2022). MANF is upregulated under ER stress as a regulatory mechanism to sustain ER homeostasis. The expression of MANF is dynamically controlled under metabolic disorders, such as obesity, non-alcoholic fatty liver disease and type-1 and -2 diabetes mellitus (Tang et al., 2022). As an extracellular factor, MANF associates to receptors, including the KDEL and NPTN receptors, or it undergoes endocytosis through binding to membrane sulfatide, a process that contributes the enhancement of ER homeostasis and downregulation of inflammation (Tang et al., 2022). Both Caenorhabditis elegans and human MANF bind sulfatide, an association that is required to protect cells from apoptosis (Bai et al., 2018). The MANF N-terminal saposin-like fold domain is a lipid-binding domain (Parkash et al., 2009) that binds sulfatide as deduced from limited trypsin proteolysis and mass spectrometry analyses (Bai et al., 2018). However, mutation of MANF at α-helical K112 (K112L), downstream of the saposin-like domain, reduces sulfatide binding (Bai et al., 2018). Nonetheless, structural studies are required to demonstrate that such a drastic mutation does not alter the overall structure of MANF. Although the sulfatide-binding site in MANF has not been characterized yet, analysis of the human sequence led to the identification of a potential binding site for the sphingolipid at a region comprising residues 29–55 within the MANF saposin-like domain (Figures 2A,D), in agreement with that observed by Ma and colleagues (Bai et al., 2018). This α-helix-loop-α-helix region contains one tyrosine (Y36) and one arginine (R39), which are both critical for sphingolipid binding in α-synuclein (Fantini and Yahi, 2011) and Dab2 (Song et al., 2020). Nonetheless, to establish whether this MANF region really does bind sulfatide needs further investigation.

### 3.2 Protein-bound sulfatides exposed for interaction with other proteins

#### 3.2.1 Toll-like receptor 4-myeloid differentiation factor-2 complex

**FIGURE 3:**
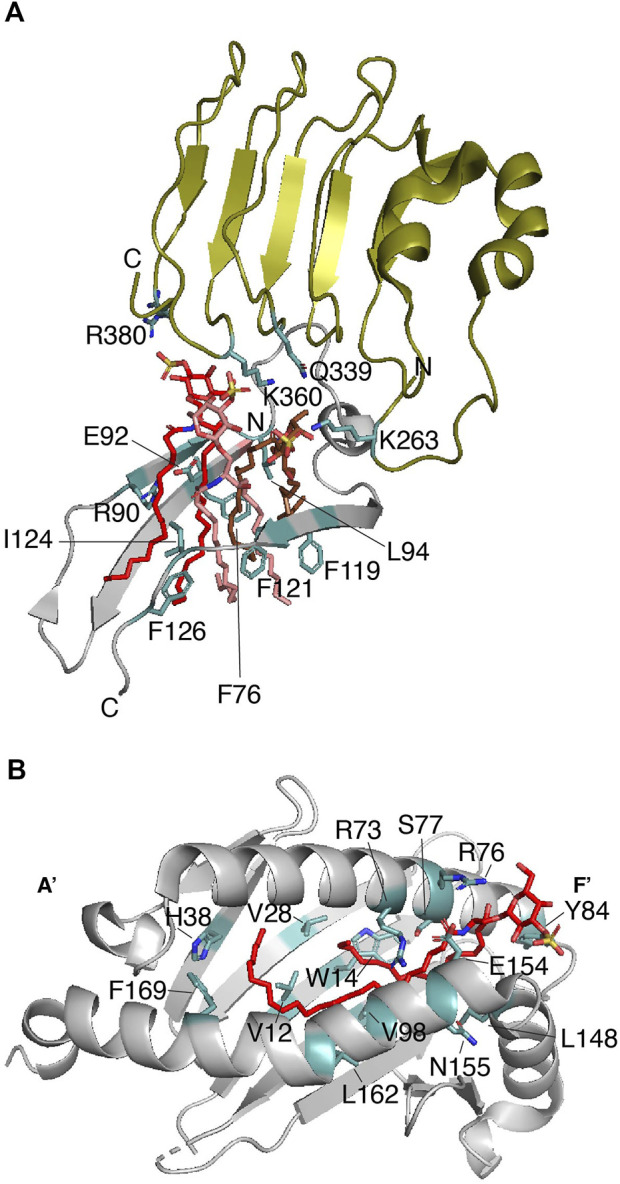
Sulfatide binding protein complexes that require a second protein for signaling. **(A)** Enlarged representation of one of the mTLR4-MD-2 dimers in complex with three sulfatides (PDB: 7MLM). mTLR4 (residues 261–383) is displayed in olive, MD-2 (residues 74–129) is in gray, and sulfatide molecules are in red, salmon, and brown. Key sulfatide-interacting residues of mTLR4 and MD-2 are colored in light teal. **(B)** The sulfatide-binding groove in the CD1a-γδ TCR complex (PDB: 7RYN). Enlarged representation of the CD1a (gray; residues 7–184) in complex with sulfatide (red). Key CD1a-interacting residues, as deduced from (Zajonc et al., 2003) are colored in light teal.

It remains unclear whether sulfatide promotes or blocks inflammation and autoimmunity. Unlike GalCs, sulfatide stimulates monocyte expression and secretion of cytokines (Constantin et al., 1994). Type II natural killer T (NKT) cells are the best characterized immune cells that promote a dominant immune regulatory mechanism in a sulfatide-dependent manner (Marrero et al., 2015). Interestingly, the length of the sulfatide fatty acid chain seems to be relevant for the innate immune response-related nuclear levels of p65 and p50 NF-kβ in antigen-presenting cells (Altomare et al., 2011). The innate immunity modulator Toll-like receptor 4 (TLR4) can be activated by the C24-sulfatide of dendritic cells (Buschard et al., 2012) but this long fatty acid chain isoform impairs lipopolysaccharide (LPS)-mediated localization of TLR4 in lipid drafts, supporting the anti-inflammatory role of sulfatide (Kim et al., 2020). Gram-negative bacterial LPS or toxins activate TLR4 and this activation occurs in a myeloid differentiation factor-2 (MD-2)-dependent manner (Shimazu et al., 1999). The crystal structure of the mouse TLR4 (mTLR4)-MD-2 complexed with C16-sulfatide gives important insights about the lipid’s binding mode and the potential identification of a consensus sulfatide-binding site (Su et al., 2021). Sulfatide promotes the dimerization of the mTLR4-MD-2 dimer (Table 1), adopting a conformation similar to that reported for TLR4-MD-2 associated with lipid A (the active moiety of LPS) and neoseptin-3 (Park et al., 2009; Wang et al., 2016). Three C16-sulfatide molecules are located at the hydrophobic pocket of MD-2 in each mTLR4-MD-2 heterodimer; the hydrophilic regions of the three sulfatide molecules are found outside of such a pocket (Figure 3A). MDS of the dimeric mTLR4-MD-2 dimer in complex with six sulfatide molecules provides additional details about the contacts (Su et al., 2021). Out of six sulfatide chains, five are buried by forming hydrophobic contacts with MD-2. The remaining lipid chain is located at the interface between mTLR4 and MD-2. In line with was described for the role of a conserved arginine residue of Dab2 (R42) for interactions with the sulfatide head group, mTLR4 R380 directly contacts the sulfate group of one sulfatide (Figure 3A) (Su et al., 2021). In addition, hydrogen bonds between the sulfate group of a second sulfatide molecule and the backbone amine and carbonyl groups of mTLR4 S386 are also observed from MDS despite the fact this residue is away from the sulfatide binding pocket. Many hydrogen bonds are formed between C16-sulfatide and mTLR4 and MD-2. For example, MD-2 R90 makes hydrogen bonds with a fatty acid chain of one sulfatide molecule, consistent with that observed with the role of the basic residues of Dab2 SBM for binding to sulfatide hydrophobic tails (Song et al., 2020). A hydrophobic loop region in MD-2, known as the F126 loop, which is located at the interface of the two mTLR4-MD-2 heterodimers, plays a major role in sulfatide-mediated function as mutations in the F126 loop drastically alter the response of HEK293T cells to mTLR4-MD-2-mediated sulfatide stimuli (Su et al., 2021). Mutations in other MD-2 residues, including F76, L94, and F119, which form the hydrophobic pocket (Figure 3A), cause a similar effect. In α-synuclein and Dab2, the equivalent aromatic residues, Y39 and Y38, respectively, may play a similar critical function (Fantini and Yahi, 2011; Song et al., 2020). In addition to the head group, the chain length in sulfatide shows differences in binding and conformational states as well as cellular responses, with shorter fatty acid chains being the preferred ligand for the mTLR4-MD-2 complex (Su et al., 2021). Curiously, sulfatide serves as an antagonist for human TLR4 but retains the preference for shorter fatty acid chains (Su et al., 2021); however, it is possible that other yet to be measured sulfatide subspecies can interact and activate the human TLR4-MD-2 dimer.

#### 3.2.2 Cluster of differentiation 1

Initiation of an adaptive immune response in vertebrates depends upon the activation of T-cells through the recognition of foreign antigens by T-cell receptors (TCRs) (Al-Aghbar et al., 2022). Activation of these receptors relies on the presence of antigen-presenting proteins located at the surface of professional cells. In addition to the major histocompatibility complex (MHC) proteins that present peptides as antigens, another group of proteins, including the paralog cluster of differentiation 1 (CD1) and the MHC class I-related molecule 1, are involved in the presentation of antigenic lipids and lipoproteins to T-cells (Pishesha et al., 2022). The CD1 proteins are grouped based on what cells they activate: group 1, which includes CD1a, CD1b, and CD1c display antigens to cytotoxic CD8^+^ T-cells, whereas the group 2, which is represented by CD1d, presents antigens to the invariant natural killer (iNK) T-cells (Moody et al., 2005).

CD1 proteins are composed of three extracellular domains (α1, α2, and α3), a transmembrane domain, and a cytosolic region. Two α-helices (α1 and α2), on top of a β-sheet, build a lipid-binding groove that is responsible for presenting the antigen to iNK T-cells (Zeng et al., 1997). The hydrophobic nature and conformation of the lipid-binding groove allows interaction with the sphingolipid tails (Barral and Brenner, 2007), leaving the polar head groups pointing away, thus, favoring their recognition by TCRs (Moody et al., 2005). For example, CD1a proteins display foreign lipoproteins such as the mycobacterial didehydroxy-mycobactin (Zajonc et al., 2005) or they can present cellular lipids such as sulfatide (Zajonc et al., 2003). Unlike what is observed in γδ TCRs (Wegrecki et al., 2022), binding of sulfatide to CD1a abolishes the αβ T-cell response due to a local conformational change in the protein (Cotton et al., 2021). The lipid-binding groove of CD1a is characterized by the presence of a double hydrophobic compartment cavity termed the A′ and F′ pockets with the A′ role being to present the lipid antigen to TCRs (Zajonc et al., 2005). In the case of the CD1a-sulfatide complex, the sulfatide displays an S-shaped structure in which the CD1a A′ pocket makes contacts with the C_18_ sphingosine backbone of the sphingolipid, whereas the sulfatide acyl chain comes out from the A′ pocket and makes contacts with the F′ pocket (Figure 3B) (Zajonc et al., 2003). The sulfatide head group forms hydrogen bonds with the charged residues R73 and S77 on the galactose head group and the sulfate moiety makes contacts with residues R76 and E154 (Figure 3B) (Zajonc et al., 2003). Thus, as observed in other sulfatide-binding proteins, an arginine residue in CD1a is required for recognition of the sulfate group of the sulfatide. As identified in other sulfatide-binding sites, a tyrosine residue (Y84) faces the sulfatide head group, whereas H38 makes contacts with one of the acyl chains of the sphingolipid (Figure 3B). Whereas CD1a serves as a sulfatide-independent ligand for TCRs, the CD1-γδ TCR can also complex sulfatide with a *K*_D_ ranging between 10–22 μM (Table 1) (Wegrecki et al., 2022). The crystal structure of CD1-γδ TCR-sulfatide reveals that a CD1a-bound sulfatide head group emerges from the F′ portal and is about 13Å from the TCR γ subunit, which it might explain why sulfatide does not inhibit γδ TCR binding to CD1a (Wegrecki et al., 2022). The sulfatide-binding site in TLR-bound CD1a is consistent with that reported earlier for the CD1a-sulfatide complex (Figure 3B) (Zajonc et al., 2003).

Cellular sulfatide is also recognized as a self-antigen by CD1d, an association that is required to activate type II NK T cells (Jahng et al., 2004). Sulfatide promotes proliferation and expansion of memory in T cells (Iwamura et al., 2012). In the central nervous system, the number of T cells that are highly reactive to sulfatide are increased and CD1d is upregulated in individuals with autoimmune encephalomyelitis (Jahng et al., 2004; Halder et al., 2007). The structural basis by which TCR interacts with the CD1d-sulfatide complex depends upon the cell type; TCR from type II NKT cells (XV19 hybridoma) orthogonally associates above the A′ pocket of CD1d in the CD1d-sulfatide complex, whereas the type I NKT TCR interacts with the CD1dF′ pocket (Girardi et al., 2012; Patel et al., 2012). As observed in the CD1a-sulfatide complex, the CD1d-C24:1 sulfatide complex shows the lipid acyl chain in the A′ pocket, whereas the sphingosine chain binds with the F′ pocket, exposing the sulfated head group at the CD1d surface (Zajonc et al., 2005). Interestingly, the A′ pocket is the most conserved region among CD1 proteins, in which the alkyl chain of the sulfatide interacts at this site by association to residues F70 and V12 (Zajonc et al., 2003).

A similar structure was reported for human γδ TCR association to the CD1d-sulfatide complex, in which the complementary-determining region (CDR) 3δ of the TCR δ-chain is responsible for contacting the sulfatide head group by forming electrostatic interactions with R103, likely providing sulfatide specificity (Luoma et al., 2013). Surprisingly, human, but not mouse, iNKT cell TCR associates with the CD1d-sulfatide complex with a *K*_D_ of 19–26 μM (Table 1) (Stax et al., 2017). Molecular modeling of the ternary complex suggests that human CD1d W153 contributes to moving the galactose moiety of the sulfatide into an orientation that energetically favors TCR binding through its CDR loops. Since W153 is replaced by G155 in mouse CD1d, the lack of a tryptophan residue at the sulfatide binding site of mouse CD1d may explain the inability of TCR to recognize the sulfatide in the CD1d-sulfatide complex (Stax et al., 2017). An alternative model, in which the sulfatide galactose head is rotated 180°, shows that the sulfate group of the sulfatide contacts a TCR CDR loop and forms electrostatic interactions with residues H68 and R95 of human CD1d, but the distance of CD1d W153 with respect to the TCR CDR loop remains the same (Stax et al., 2017).

### 3.3 Proteins that cage sulfatides

#### 3.3.1 Glycolipid transfer protein

**FIGURE 4:**
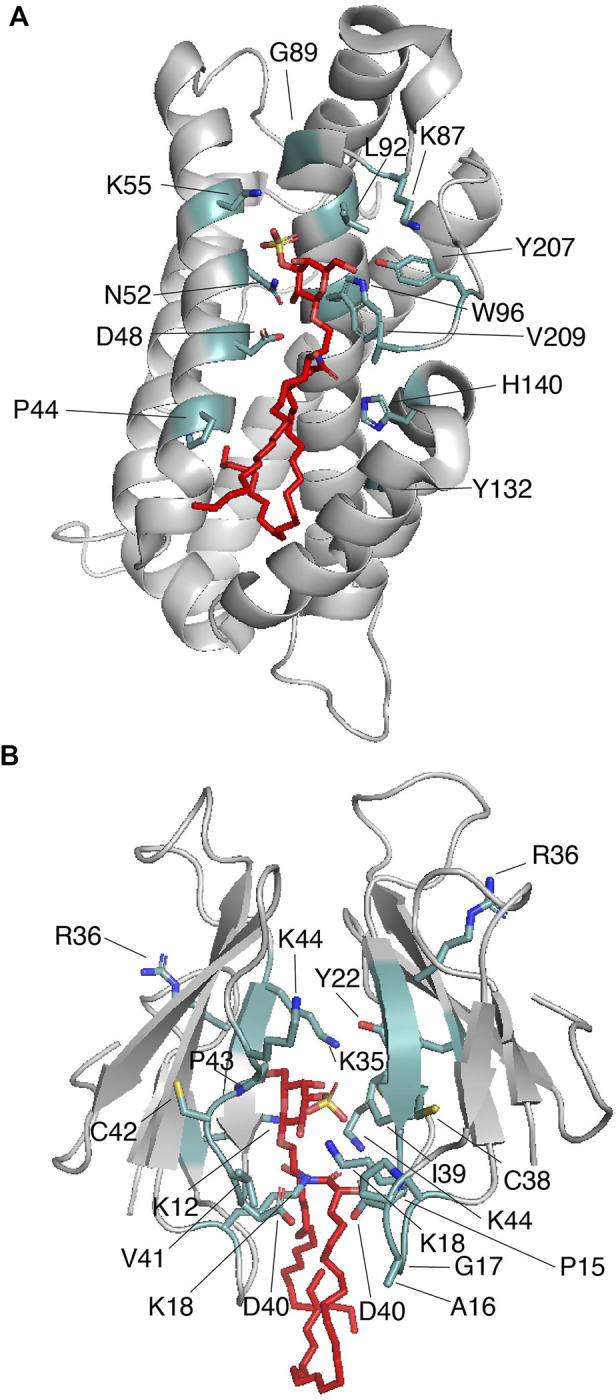
Proteins that cage sulfatides. **(A)** Human GLTP (gray) in complex with sulfatide (red; PDB: 3RZN). Key sulfatide-interacting residues are colored in light teal. **(B)** Two molecules of Taiwan cobra cardiotoxin A3 (gray) complexed with sulfatide (red; PDB: 2BHI). Key sulfatide-interacting residues are colored in light teal.

Intermembrane transfer of sphingolipids can be enhanced by glycolipid transfer proteins (GLTPs), which are α-helical sensors of glycolipid levels that target the plasma membrane and the ER (Mishra et al., 2020). GLTPs (∼24 kDa) display head group selectivity and associate with most of the nonpolar hydrocarbon chains of the ceramide region of the sphingolipid through their hydrophobic pocket (Malinina et al., 2004). Indeed, the requirement of both sphingolipid regions is evident from studies in which the free head groups do not impact GLTP activity (Abe et al., 1982). The GLTP structural topology, a two-layer orthogonally bundled fold (Figure 4A), favors the ceramide moiety to properly interact with the hydrophobic pocket of the protein, which is collapsed in the sphingolipid free state. Sphingolipid binding engages dimerization of GLTP (Table 1), which may be crucial for controlling GLTP activity (Samygina et al., 2013). In human GLTP, orientation of the ceramide moiety in the hydrophobic pocket is driven by hydrogen bonds formed with residues D48 and H140 and stabilized by interaction with Y132 (Figure 4A). GLTPs display two sphingolipid binding modes (Malinina et al., 2006): 1) the “Sphingosine out” mode, in which the sphingolipid undergoes a bend at carbon 6 of the sphingosine chain, forcing it to project away from the hydrophobic pocket; and 2) the “Sphingosine in” mode, in which both the sphingosine and acyl moieties are located in the GLTP hydrophobic pocket (Malinina et al., 2015). Crystal structure studies of human GLTP and a GLTP D48V mutant in association with sulfatide (C24:1) show additional insights into the interaction (Samygina et al., 2011). In the wild-type GLTP, sulfatide is bound in the sphingosine-out mode, and the overall conformation is very similar to that of the galactosylceramide-bound state (Malinina et al., 2006). Residues D48, N52, K55, W96, H140, and Y207 form hydrogen bonds with the sulfatide head group with K55 interacting with the sulfo-group (Figure 4A) (Samygina et al., 2011). The residue P44 plays an important role in sulfatide-mediated dimerization by forming van der Waals contacts with the sphingosine chain (Figure 4A) (Samygina et al., 2011). A D48V mutation favors GLTP transfer selectivity to sulfatide and switching into the *“sphingosine in”* mode. By introducing the D48V mutation in GLTP, the sulfated galactosylceramide group of the sulfatide makes contact at the entry portal, facilitating insertion of the acyl chain within the upper compartment of the hydrophobic pocket, followed by entry of the sphingosine chain at the same site, leading to the *sphingosine-in* binding mode (Samygina et al., 2011). Comparison of the glycosphingolipid-free and bound states of GLTP indicates that, unlike the head group binding site, conformational changes occur at the hydrophobic contacts (Malinina et al., 2015), which may explain the broad range of sphingolipid ligands for the protein.

There is strong evidence that the membrane binding region of GLTP overlaps, or it is nearby, the glycolipid-binding site of the protein [reviewed in (Malinina et al., 2015)]. In addition to tryptophan and tyrosine residues, the membrane-binding site involves multiple lysine residues that may help to properly orient the protein towards the head group of the lipid. Alternatively, it is possible that the long hydrocarbon chain of the lysine residues is required to make contact with the hydrophobic tails of the sulfatide, as occurs with that described for sulfatide recognition by Dab2 SBM (Song et al., 2020).

#### 3.3.2 Cardiotoxin

The Naja atra Taiwanese cobra β-structured cardiotoxin A3 (CTX A3) belongs to a family of positively charged toxins that can induce severe necrosis in tissues and systolic heart arrest in envenomed individuals (Kini and Koh, 2020). CTX A3 binds to the plasma membrane at sulfatide-enriched domains and is internalized through a formation of a pore in the host cell membrane (Wu et al., 2012) targeting mitochondria (Wang et al., 2005). The crystal structure of CTX A3-sulfatide in detergent micelles shows that the peptide requires interaction of both the sulfatide head group and the ceramide region, promoting CTX A3 dimerization (Figure 4B; Table 1) (Wang et al., 2006). Unlike what is observed in the CD1 (Zajonc et al., 2003; Wegrecki et al., 2022) and GLTP (Samygina et al., 2011) proteins, the sulfatide head group is buried in a groove formed by the CTX A3 dimer with the acyl tails pointing slightly away from the protein structure (Wang et al., 2006). On one of the CTX A3 monomers, the sulfatide galactose moiety forms hydrogen bonds with the amino groups of K12 and K18 and the carbonyl oxygen group of R36 and C38, whereas the sulfatide sulfate group forms a hydrogen bond with the amino group of K35 (Figure 4B) (Wang et al., 2006). On the second CTX A3 monomer, Y22, R36, G37, C38, I39, and K44 accomodate the sulfatide head group to the protein surface with the K44 residue stabilizing sulfatide bending (Wang et al., 2006). In the CTX A3-sulfatide crystal structure, the sphingosine chain of the sulfatide comes in contact with the detergent, whereas the fatty acid tail inserts into the CTX A3 dimer (Wang et al., 2006). The acyl tails of the sulfatide makes contacts with the D40-K44 region in one monomer and the P15-K18 and I39-D40 regions in the second monomer. Thus, as observed in Dab2, lysine residues are involved in hydrophobic interactions with the lipid. Additional NMR studies indicate that the sulfatide head group changes from a bent shovel to an extended conformation upon binding to CTX A3 (Tjong et al., 2007). This conformational change is suggested to increase the affinity between CTX A3 dimers with a reorientation of the resultant tetramer, which may trigger pore formation in the host plasma membrane (Wang et al., 2006; Tjong et al., 2007).

## 4 Concluding remarks

Several groundbreaking studies have revealed that membrane sulfatide facilitates a wide range of cellular functions using recognition mechanisms with a remarkable degree of heterogeneity. Given the localization of sulfatide at the outer plasma membrane, one common function is to serve as a receptor for cell-surface proteins. The high-resolution of the protein-sulfatide structures provide some comparative information about how specific a sulfatide head group is or whether the ceramide portion of the sphingolipid drives protein activity. The consensus structural data indicates that it is likely that the side chain of an arginine, and in some cases lysine, makes electrostatic contact with the sulfate moiety (Zajonc et al., 2003; Wang et al., 2006; Samygina et al., 2011; Stax et al., 2017; Song et al., 2020). It is possible that a nearby tyrosine residue plays a critical role in this interaction, but it is unlikely that it provides the specificity as observed for the wide range of the α-synuclein (Fantini and Yahi, 2011) and CD1a (Paterson et al., 2021) sphingolipid interactions. Other sulfatide-binding proteins primarily contact the ceramide region with a specific fatty acid chain length, and degree of unsaturation and hydroxylation. In mammals, there are about fifty different isoforms of ceramide, and, therefore sulfatides, reflecting these structural differences (Shaner et al., 2009; Mirzaian et al., 2015). This heterogeneity is cell- and tissue-specific and age-related in humans (Ando, 2014; Pradas et al., 2018). As suggested by Zajonc and colleagues (Zajonc et al., 2003), and considering the generally observed moderated affinity for sulfatide (Table 1), it is possible that sulfatide-binding sites can serve as a ruler to discriminate the length of an acyl chain and exclude those that the number of carbons impair proper binding leading to very weak affinity for the sphingolipid. Nonetheless, the ceramide portion of the lipid may have impacts in biological processes such as neurodegeneration, diabetes, differentiation, myelin stability, aging, and host-pathogen interactions (Yuki et al., 2011; Buschard et al., 2012; Hirahara et al., 2017; Pintado-Sierra et al., 2017; Gil et al., 2019; Williams et al., 2020; Su et al., 2021).

There are major challenges that hopefully will be resolved through future research efforts such as how sulfatide signals, identification of external cues that direct either sulfatide synthesis or turnover, the contribution of circulating sulfatide in body fluids under different metabolic states and the impact on lipid rafts, high-resolution technology to visualize individual sulfatide molecules, the existence of protein partners that enhance and stabilize protein-sulfatide interactions, and the elucidation of the specificity for the sulfatide head group and/or for the ceramide moiety. Since the affinity for sulfatide-binding proteins is in the micromolar range (Table 1) and that sulfatide levels may form lateral membrane domains (Bjorkqvist et al., 2008), it will be important to establish the spatiotemporal distribution of the lipid under a diverse range of stimuli. Elucidating how sulfatide-binding proteins work in concert with enzymes involved in sulfatide synthesis and degradation will also be crucial for understanding the role of sulfatides in physiological and disease conditions. Using rapidly expanding high-resolution images of biological membranes will provide additional insights of the distribution of membrane sulfatide, regulatory processes, and, in the end, offer new breakthroughs in our current understanding of protein-sulfatide interactions and signaling.

## Abbreviations

ARSA, arylsulfatase; CD1, Cluster of differentiation 1; CGT, ceramide galactosyltransferase; CST, cerebroside sulfotransferase; CTX, Cardiotoxin; Dab2, Disabled-2; GalC, galactosylceramides; GLTP, Glycolipid-transfer protein; MANF, Mesencephalic astrocyte-derived neurotrophic factor; MD-2, Myeloid differentiation factor-2; MLD, metachromatic leukodystrophy; NKT, natural killer T; SBD, sphingolipid-binding domain; SBM, sulfatide-binding motif; TCR, T-cell receptor; TLR4, Toll-like receptor 4.
